# Dispersion behaviour of insoluble particles with different surface properties in non-aqueous media – biopolymer based oleogels

**DOI:** 10.1039/d5sm00596e

**Published:** 2025-08-29

**Authors:** Megan Holdstock, Brent Stuart Murray, Anwesha Sarkar, Paraskevi Paximada, Michael Rappolt, Isabel Celigueta Torres

**Affiliations:** a Food Colloids & Bioprocessing Group, School of Food Science & Nutrition, University of Leeds LS2 9JT UK b.s.muray@leeds.ac.uk; b National Alternative Protein Innovation Centre (NAPIC) UK; c Nestlé Product Technology Centre York YO31 8FY UK

## Abstract

Particles with some degree of hydrophilicity are known to aggregate when directly dispersed in non-aqueous media. Proteins are generally insoluble in oil and have complex surface properties, but they may form networks in oil like more simple colloidal particles, depending on particle size and surface hydrophilicity. Here, the particle size of pea protein isolate (PPI) particles in oil was reduced to submicron sizes by stirred media milling. The rheology of milled PPI oil suspensions was compared to dispersions prepared with two types of colloidal silica particles – hydrophobic and hydrophilic. PPI particles form structured aggregates in oil which break down under shear that, similarly to silica, can form an elastic network like an oleogel system. As PPI size decreased, aggregation increased, shown by higher apparent viscosities and gel strength. PPI particles with an average size of 1 μm exhibited elastic behaviour (*G*′ > *G*′′) at 11.2 wt%. Rheological scaling models obtained two fractal dimensions: a higher intra-floc dimension and a lower network backbone dimension, suggesting that colloidal PPI and silica particles have an inhomogeneous microstructure with denser particle flocs compared to a relatively sparse backbone. For smaller PPI particles the inter- and intra-floc fractal dimensions become like that of hydrophobic silica, suggesting that the average ‘surface’ character of the PPI may be close to that of the silica. Therefore despite the complexity of the protein surface, parallels can be drawn with simpler colloidal systems. Pre-wetting the particles with ethanol tuned this behaviour, highlighting the role of surface chemistry in gel formation.

## Abstract 2

Illustration of the similarities and differences between dispersions of silica and plant protein particles as oil-structuring agents.

## Graphical abstract

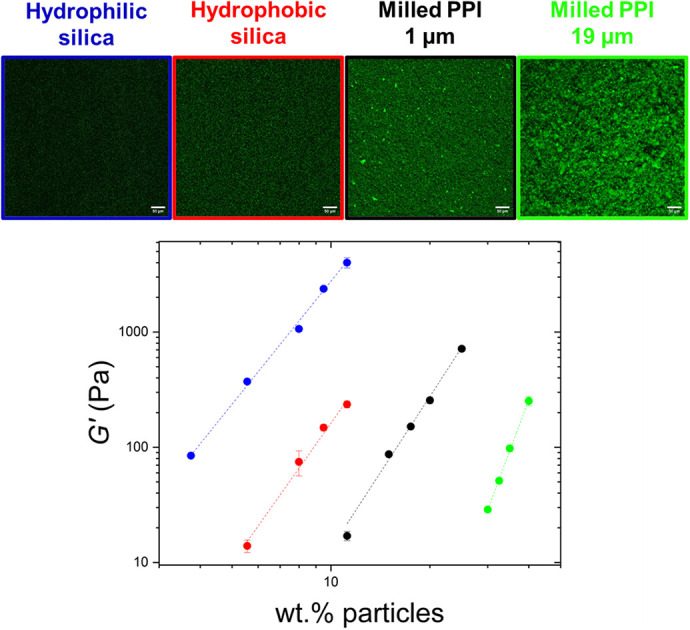

## 1. Introduction

Non-aqueous dispersions are relevant to a wide range of applications such as paints, coatings, drilling fluids and also various confectionery products. Controlling the dispersion of solid particles in less polar media, such as oils, poses a considerable challenge, particularly when the particle is amphiphilic in nature. Although milling techniques and surface modifications are highly developed for the preparation of such non-aqueous dispersions, direct dispersal of polar or even amphiphilic particles into non-polar media remains a difficult task.

Interactions between particles occurs as they encounter each other *via* Brownian motion and/or shear induced collisions. Attractive van der Waals forces exist between particles, dependent on the interparticle distance and the nature of the particles.^1^ However, other attractive forces can also exist between polar particles, for example hydrogen bonding, leading to particle aggregation and agglomeration.^2^ The formation of a complex driven by hydrogen bonding is thermodynamically more favourable in non-polar media. This is due to the lack of competing hydrogen bonds and the low dielectric constant of non-polar solvents, driving the association of particles with the propensity to accept and donate hydrogen bonds.^3^ Techniques that induce electrostatic repulsion and/or steric stabilisation are typically used to combat the attractive forces between colloidal particles in aqueous solution. In non-polar environments it is difficult to generate charges on the surface of solid particles because non-polar solvent molecules do not associate sufficiently strongly with surface charges to form a tight solvation shell, thus it is easy for oppositely charge ions to recombine.^4^ However, additives (“dispersants”) that strongly adsorb to the surface can impart a steric barrier to combat attractive forces. The effectiveness of a dispersant is dependent on its chemical structure in terms of the balance between its tendency to adsorb or aggregate in the bulk nonpolar solvent, as well as its conformation at the surface – larger moieties that protrude away from the surface in a good solvent impart greater steric repulsion. The effectiveness of a surfactant may be limited for particles that possess both hydrophilic and hydrophobic regions, since adsorption can occur in multiple orientations, head and/or tail down, resulting in minimal changes in interparticle interactions overall.

If dispersion *versus* aggregation of the polar particles in the non-polar medium can be controlled, this opens up the very useful possibility of manipulating the viscosity (*η*) and/or the gelation of various non-aqueous media. The term oleogel has been coined relatively recently for gelling oil systems and is the subject of much current research, where tunable viscoelasticity of a single oil type is desired, often obtained by adding particles forming networks in the oil phase.^5–13^ At the same time, many existing formulations of oil continuous systems may contain polar particles, or polar particles may be generated within them, and it is necessary to control the particle aggregation and hence the viscoelasticity of the formulation as a whole. In many oleogel applications (in pharmaceuticals, agrochemicals and person care, for example) it would be a distinct advantage if the particles were completely biodegradable. This also applies to various existing foodstuffs, but with the added attraction that anything that replaces some of the oil content can potentially make the product healthier in terms of reducing the high calorie contribution from lipids, as well as introducing novelty in terms of improved health^14^ or organoleptic properties.

The most widely studied model system of non-aqueous dispersions is silica particles in various oils.^15–23^ Silica particles can be synthesized *via* various methods to achieve well-controlled primary particle sizes ranging from a few nanometres to microns. Silica powders can therefore have very high specific surface areas which makes the surface chemistry a crucial factor for their dispersion. The silica surface is inherently hydrophilic, comprising of siloxane and silanol groups. This surface chemistry lends itself to various types of chemical modification, such as through the adsorption of cations, esterification of silanol groups or alkylation with organosilanes, that change the particle wettability.^15,24^ However, addition of silica is not appropriate for many applications that come into contact with the human body. An alternative type of particle that would be compatible with most consumer products is insoluble protein particles. Although there is growing consumer perception that foods with increased protein content are healthier,^25,26^ relatively little is known about how such sparsely soluble proteins or protein aggregates behave in a non-polar environment. There have been some studies on the use of proteins to formulate oleogels,^7,8,10,27–29^ but there is limited understanding of how the addition of particles with different surface properties may affect their dispersion in oils.

The challenge of using proteins and their aggregates as particles to influence the oil viscoelasticity is, of course, that proteins are vastly more complex than inorganic particles such as silica, with or without silica surface modification. For simplicity, throughout the rest of this discussion we will simply refer to entities termed ‘proteins’, when in fact these proteins will almost always be aggregates of many protein molecules in non-aqueous media. The outer surface of these proteins, if such entities can be considered to have a definite surface, is typically hydrophilic overall, but the complex structures of proteins means that there will be a complex distribution of both hydrophilic and hydrophobic surface regions. These groups can give rise to a range of different attractive or repulsive interactions. Initially, however, overcoming attractive interactions to disperse such particles into a hydrophobic environment in the first place is challenging and so far most previous protein oleogel systems have been created using indirect approaches. Such methods start with proteins in an aqueous dispersion to formulate systems like emulsions, foams, hydrogels, and microgels.^8,27,29^ These can subsequently be converted to oleogels by drying or solvent exchange. Although effective, these techniques can be laborious and require high amounts of solvent, limiting their application as an industrial process and with consumer risk of solvent exposure.

Consequently, the properties of protein particles dispersed *directly* into an oil environment remains largely unaddressed in literature and therefore the aim of this work was to investigate the dispersion behaviour of protein particles added directly to oil and to compare the behaviour with the more well-characterized system of silica particles of known surface chemistry. A plant-derived protein − pea protein − was selected on account of its limited aqueous solubility (∼30%^30^), a characteristic typical of plant storage proteins. This is typically attributed to its high surface hydrophobicity and larger particle size.^28,31,32^

The systems were characterized largely *via* rheology, along with light scattering techniques for measurement of particle size and microscopy. A range of mechanical treatments (stirred media milling) was used to vary the size of protein particles, from mm to the submicron range. Rheological scaling models were applied to try and evaluate the microstructure of the particle networks and relate this to the observed macroscopic rheology properties. To our knowledge this is the first study that details non-aqueous, non-modified protein particles in hydrophobic media acting as rheology modifiers, which might have significant applications in soft matter and allied areas.

## 2. Materials and methods

### 2.1 Materials

Pea protein isolate (PPI, Nutralys S85F) containing 83.8% protein was supplied by Roquette (Lestrem, France). Magnesium chloride salt was obtained from Sigma-Aldrich (Gillingham, UK). Silica particles with different hydrophobicity, characterized as having 100 and 35% of the natural surface density of silica SiOH groups were a gift from Professor Binks, University of Hull, previously obtained from Wacker-Chemie GmbH (Munich, Germany). In the case of the 35% SiOH particle, the surface was treated with dichlorodimethylsilane and the relative silanol content determined by acid–base titration. The nominal particle size of the silica particles was 20 nm. PPI and silica particles were placed in a desiccator over a magnesium chloride saturated salt solution and equilibrated to room temperature for at least 5 days before use. This salt solution created an environment with a controlled relative humidity of 33%. Sunflower oil was purchased from a local supermarket and used without purification. Ethanol (≥99.8%, analytical grade) was supplied by VWR Chemicals (Lutterworth, UK).

### 2.2 Methods

#### 2.2.1 Preparation of protein suspensions

Stirred media milling (SMM) was explored as a method to reduce the reduce the size of PPI particles in oil. SMM consists of a rotating agitator and grinding media within a chamber generating shear, impact, and frictional forces to break aggregates towards the primary particle size.^32^ Two milling apparatus were used. The first type consisted of a stainless steel grinding chamber (of volume 365 mL excluding spindle) plus a ceramic spindle that were fabricated for use with a Silverson high shear mixer (L5M-A, Silverson, Chesham, UK). The mill was operated with a 0.5 grinding media to fill ratio, using yttrium-stabilised zirconium oxide milling beads of 600–800 μm diameter. Suspensions of volume 75 mL were transferred to the grinding chamber and the spindle set to 100 rpm for 1 min to allow the suspension to coat the milling beads. The mill was then operated at the 4000 rpm and the suspension milled for 60 to 240 min. Suspensions were first milled at 10 wt% to investigate the effect of milling time on particle size. For rheological analysis, PPI-oil suspensions were milled at 25 wt% and subsequently diluted with sunflower oil to obtain lower protein concentrations. To reduce the temperature increase during milling, milling was carried out in 15 min intervals with a 5 min pause between cycles, and ice was placed around the milling chamber.

Milling was also conducted at Nestlé Product Technology Centre, York using industrial scale apparatus. A Weiner ball mill (Royal Duyvis Wiener B.V., Koog aan de Zaan, Netherlands) was operated with a 0.68 grinding media to fill ratio, using chrome steels balls of diameter 9.5 mm. Suspensions of 1 kg at a protein concentration of 50 wt% were transferred to the grinding chamber and milled at a speed of 219.4 rpm for two hours. Desired protein concentrations were achieved by dilution with sunflower oil.

This second method was used to prepare larger volumes and higher particle concentrations of milled PPI, that were then diluted to the desired protein concentration. This method generated a suspension of PPI particles in oil with volume weighted mean diameter (*D*_4,3_) = 19 μm, as detailed in Section 2.2.5.

Since the mechanisms of the two milling methods are identical and the particle size distributions produced by both processes were comparable (Fig. S1), the only distinction was that the first method achieved a similar particle size distribution (PSD) in shorter times due to the higher speed and smaller ball size, increasing the frequency of stress events. The results from both methods can therefore be compared.

In samples where a pre-wetting step was used, the PPI particles were added to an excess of ethanol, a sufficient volume to fully submerge and suspend the particles, and continuously stirred for 10 min. The particles were then left at room temperature for three days to allow excess solvent to evaporate. After ethanol evaporation, a powder remained, which was then ground down with a pestle and mortar and then milled as above.

#### 2.2.2 Preparation of silica suspensions

The silica powders were dispersed in sunflower oil using a rotor stator homogenizer (Ultra Turrax, T25, IKA Werke, Germany) at 13 500 rpm for 4 min. Dispersions were then placed under vacuum to remove bubbles. In samples where a pre-wetting step was used, the silica particles were added to an excess of ethanol, a sufficient volume to fully submerge and suspend the particles, and continuously stirred for 10 min. The particles were then left at room temperature for three days to allow excess solvent to evaporate. After ethanol evaporation, a powder remained, which was then ground down with a pestle and mortar and then milled as above.

#### 2.2.3 Microscopy

Scanning electron microscopy in the Leeds Electron Microscopy and Spectroscopy Centre (LEMAS) was used to image the dried PPI powder. The sample was sputter coated with gold and imaged using a Carl Zeiss EVO MA15 SEM (Carl Zeiss MicroImaging GmbH, Jena, Germany) at an accelerating voltage of 20 kV.

Light microscope (Nikon Optiphot, Tokyo, Japan) images were collected with a digital camera (Leica MC120 HD, Leica Microsystems, Wetzlar, Germany). Particle suspensions were diluted before being transferred to welled glass slides and covered with a cover slip. Microscopy was performed using a 10× objective lens. Images were converted to grayscale using the image analysis software ImageJ.

PPI and silica particles suspended in oil were also stained with fluorescent dyes and imaged using a confocal microscope (Leica DM6000 CS, Leica Microsystems, Wetzlar, Germany). PPI particles were stained with Fast Green (0.2 mg mL^−1^ in ethanol) and the silica particles were stained with Acridine Orange (0.05 wt% in ethanol). The dyes were gently mixed into the sample manually before placing on the microscope slide. Laser excitation of the fluorescent samples was at 633 nm for Fast Green and 488 nm for Acridine Orange. Images were taken at 20× magnification and processed using ImageJ. Non-linear (gamma) and brightness adjustments were applied to ensure all components of the image were visible. A softening filter was also applied to reduce noise. These adjustments were made consistently across all images to improve visual clarity without altering the underlying data.

#### 2.2.4 Rheology

Rheological analysis of the silica and PPI dispersions was performed using an Anton Paar MCR 302 rheometer (Anton Paar GmbH, Graz, Austria). All measurements were performed at 20 °C, using a 50 mm diameter circular plate–plate geometry, sandblasted to avoid slip (upper plate PP50/S, lower plate Inset I-PP50/SS/S). Measurements of *η* were conducted in the shear rate (*γ̇*) range 0.01–1000 s^−1^. Amplitude sweep measurements were performed by increasing the shear strain (*γ*) logarithmically from 0.01 to 100% at a fixed frequency of 1 Hz. Measurements were performed in triplicate using a new sample loading for each measurement and the mean values are plotted in all subsequent rheology figures with the standard deviations about these means as the error bars. Data plotting and curve fitting were performed using OriginPro 2024 (OriginLab, Massachusetts, USA). Statistical analysis on the significance between data sets was calculated using analysis of variance (ANOVA) with Tukey *post hoc* test, significance level *p* < 0.05.

In the amplitude sweep measurements, the *γ* value at which the storage modulus (*G*′) had decreased by 5% from its initial value (
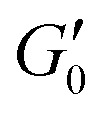
) was taken as the limit of linearity (*γ*_0_), as described by De Vries *et al.*^8^ This value (*γ*_0_) was estimated by interpolating the data to identify the strain corresponding to 95% of 
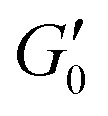
.

Although milling was effective in reducing the particle size of protein, sedimentation was still observed for the samples. However, for the milled samples used in this work, this sedimentation occurred over timescales far longer than the rheological measurement periods, with sedimentation only observed after a few days in storage.

#### 2.2.5 Particle size analysis

The particle size distributions (PSDs) of PPI particles were measured by light scattering with a Mastersizer 3000 equipped with the Hydro SM wet sample dispersion unit (Malvern Instruments, Worcestershire, UK). Suspensions were dispersed in sunflower oil until the laser obscuration reached >1%. PSDs were obtained using Mie theory as a mathematical model; the refractive index for sunflower oil was set to 1.464, and 1.54 for PPI in oil. The volume weighted mean diameter (*D*_4,3_) is calculated according to:1
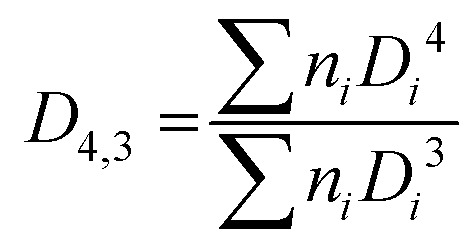
where *n*_*i*_ is the number of particles of diameter *D*_*i*_.

## 3. Results and discussion

### 3.1 Particle size

**Fig. 1:**
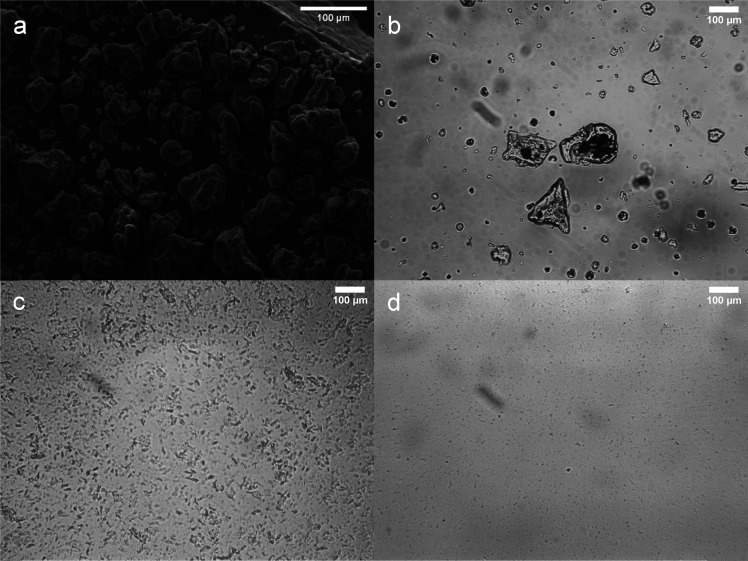
Scanning electron micrograph (SEM) image of dried PPI powder (a). Optical microscopy images of PPI particles ball milled in sunflower oil for: (b) 0 min, (c) 60 min, and (d) 240 min.

**Fig. 2:**
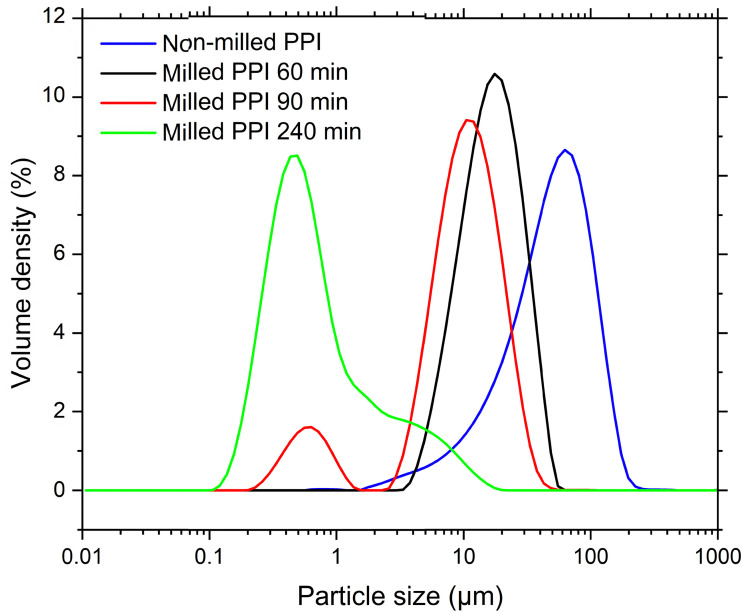
Milling time-dependent particle size distributions of non-milled and milled protein isolate particles in sunflower oil at time intervals up to 240 min at 4000 rpm stirrer speed and a particle concentration of 10 wt%.

Dry PPI particles have much larger particles sizes than any PPI in the milled dispersions, as shown *via* SEM – see Fig. 1(a). These particles are composed of aggregated protein formed during the extraction process.^32^ When dispersed in oil the protein particles remain as large particles (as shown in Fig. 2), which were poorly stable against sedimentation. After milling using the first, smaller scale mill (see 2.2.1) for 60 min *D*_4,3_ decreased from 57.0 μm to 18.4 μm and the PSD showed a monomodal peak (Fig. 2). After 90 min the PSD shifted to smaller sizes and became bimodal, indicating the presence of a submicron (0.01–1 μm) plus a larger micron-sized (1–100 μm) particle population. Increasing the milling time to 240 min led to an increase in the submicron peak area, a reduction in the micron peak area, and a shift in both peak positions to slightly smaller sizes. Milling times of 90 and 240 min decreased *D*_4,3_ to 11.2 and 1.4 μm respectively. These results show that SMM can effectively reduce the size of PPI particles in oil into the submicron region, with longer milling times expected to increase the population of PPI in this size range. Similar PSDs were reported by Li *et al.* for milling of aqueous PPI dispersions. These authors found that SMM altered the conformation and aggregation state of the proteins, generating highly water-soluble PPI particles.^32^ It is possible that SMM of PPI in an oil environment, as performed by ourselves here, also alters the surface properties of the particles, although we have no direct evidence for this yet.

The model system of fumed silica particles (hydrophobic and hydrophilic) on the other hand has a nominal size of 20 nm, although these are also known to form aggregates with sizes ranging from 100 to 1000 nm.^22,33^ The silica particles were not milled to reduce the particle size, as this aggregate size was deemed sufficient (Table 1).

**Table 1:** *D*_4,3_ values of non-milled and milled PPI particles at time intervals up to 240 min at 4000 rpm stirrer speed and a particle concentration of 10 wt%

Milling time (min)	*D*_4,3_ (μm)
0	57.0 ± 1.8
60	18.4 ± 0.2
90	11.2 ± 0.3
240	1.4 ± 0.02

### 3.2 Flow behaviour

**Fig. 3:**
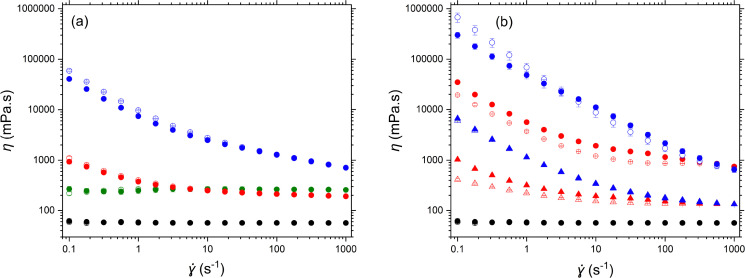
(a) Average viscosity *η* of 25 wt% pea protein particle (PPI) suspensions in sunflower oil as a function of shear rate (*γ̇*): 1 μm milled (blue); 19 μm milled (red); 57 μm non-milled (green); pure oil (black). (b) Average *η* of 4 (triangles) and 11.2 wt% (circles) fumed silica particles in sunflower oil as a function of *γ̇*: particles with 100% surface silanol coverage (blue triangles and circles, respectively); hydrophobic particles with 35% surface silanol coverage (red triangles and circles, respectively); pure oil (black circles). Increasing *γ̇* sweep = filled symbols, decreasing *γ̇* sweep = open symbols. Error bars indicate standard deviation of triplicate measurements.

The *η* of 25 wt% PPI particle suspensions of varying mean size was studied as a function of shear rate (*γ̇*) – see Fig. 3. The suspension of non-milled PPI particles exhibits Newtonian behaviour across the *γ̇* range applied. The *η* of the suspension was the same order of magnitude as that of the continuous phase. As the size of PPI particles is reduced by milling, the *η* of the system increases. Milled protein particle suspensions exhibit non-Newtonian, shear-thinning behaviour (Fig. 3(a)). The high *η* observed at low *γ̇* suggests structuring due to particle aggregation. This is more pronounced as the particle size decreases, most likely due to the increase in particle surface area and decrease in average interparticle distance. The microstructure of aggregated particles is broken down under increasing *γ̇*, leading to the observed reduction in *η*. Fig. 3(a) also shows the corresponding measurements of *η* when the *γ̇* was reduced back down to the lowest value in the same series of steps but in reverse. It is seen that there was virtually no hysteresis, indicating complete reversibility of agglomeration and break up of the particles, at least over this range of *γ̇* and time scales.

For comparison, Fig. 3(b) shows flow curves for the model silica dispersions of known surface chemistry: unmodified hydrophilic silica particles and hydrophobic silica particles with only 35% of the surface occupied by silanol (SiOH) groups. The results show that both types of silica particles exhibited non-Newtonian, shear-thinning behaviour analogous to that of the milled protein particles (Fig. 3(a)). However, the difference in % of surface silanol groups led to an order of magnitude increase in the low shear *η* of the more hydrophilic (100% SiOH) suspensions. This was expected, due to the greater propensity of these particles to aggregate *via* SiOH-mediated H-bonds when dispersed in a solvent with limited hydrogen-bonding ability. However, the high shear *η* of both types of particles were very similar, suggesting that in each case any such H-bonded structure was broken down by the mechanical forces exerted at the highest *γ̇* (*i.e.*, 200 to 10^3^ s^−1^). This again points to weak forces of aggregation, as also evidenced by the largely reversible nature of the flow curves for both particle types on decreasing the shear rate. (The hydrophobic silica shows a slight reduction in *η* after the shear cycle whereas the hydrophilic silica at 11.2 wt% shows a slight increase in *η* as a result of the cycle, which at present we cannot explain). Superficially then, both the silica particles and the much more complex protein particles behave very similarly when dispersed in oil, although the absolute magnitude of the values of *η* for the silica particle suspensions were comparable or higher than those of the PPI protein particles at lower particle concentrations, most likely due to their significantly smaller primary particle size and therefore larger specific surface contact area of the silica. Surface properties will also have an effect, with the lower viscosities of the protein systems possibly due to hydrophobicity of the particle surface, resulting in weaker particle–particle interactions.

### 3.3 Gel behaviour

**Fig. 4:**
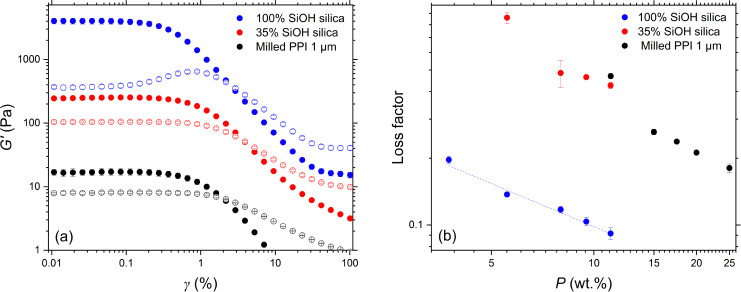
(a) Oscillatory shear strain (*γ*) sweeps of 11.2 wt% fumed silica particles and milled PPI (1 μm) dispersed in sunflower oil (*G*′ = filled symbols, *G*′′ = open symbols). (b) Loss factor as a function of particle concentration *P*. Hydrophilic silica particles with 100% surface silanol coverage (blue); hydrophobic silica particles with 35% surface silanol coverage (red); PPI (black). In (b), hydrophilic silica data are fitted to a power law model with an exponent of −0.6 with an *R*^2^ value of 0.960. Error bars indicate standard deviation of triplicate measurements.

Oscillatory rheology was conducted to gain further insight into the behaviour of the structured protein and silica aggregates in sunflower oil. Here we examined the milled PPI particles of the smallest size (*D*_4,3_ = 1 μm) and thus greatest degree of aggregation (Fig. 3(a)). It can be concluded from the initial *γ* sweeps (Fig. 4(a)) that, when dispersed at 11.2 wt%, all three particle types exhibit viscoelastic behaviour. The three particle suspensions show a plateau in both the storage modulus (*G*′) and the loss modulus (*G*′′), defining a linear viscoelastic region (LVER). The *G*′ values are higher than *G*′′ within the LVER in all three curves, *i.e.*, the loss factor (*G*′′*/G*′) < 1, demonstrating gel-like behaviour (Fig. 4(b)). Thus, the silica and milled protein particles aggregate to form structured networks with elastic character. High *γ* disrupt these networks, as shown by the moduli crossover, thereafter the response is dominated by *G*′′, *i.e.*, the suspensions flow.

The milled protein particles at the same 11.2 wt% concentration formed networks with a lower elasticity (*G*′ = 17 Pa) than either of the two types of silica particles (Fig. 4(a)). The networks formed by the more hydrophobic silica particles were an order of magnitude stronger (*G*′ = 240 Pa), whereas *G*′ of hydrophilic silica particle gel network was two orders of magnitude higher (*G*′ = 4000 Pa). It might be anticipated that the PPI particles would form a network with a strength intermediate between that of the hydrophilic and hydrophobic silica, given the amphiphilic nature of the protein surface. The reason for this not being the case (*i.e.*, lower *G*′ for the protein particles in both cases) may again be a reflection of the smaller size and thus higher specific area of the silica particles, leading to more particle–particle interactions per unit mass, presumably *via* van der Waals forces and H-bonding. Increasing the proportion of surface silanol groups from 35 to 100% certainly increased *G*′ significantly, suggesting SiOH H-bonding may be the dominant network forming force, whereas the hydrophobic modified SiOH groups will be preferentially solvated by the oil molecules. Such orders of magnitude differences have been demonstrated in mineral oil^17^ and olive oil.^22^ The surface of the protein particles is much more complex than that of silica, possessing a variety of surface groups which may reduce the number of contact potential cross-linking points per unit area. Milling of PPI in an aqueous environment has been shown to *increase* the surface hydrophobicity of the protein, which alters its dispersion behaviour.^32^ These workers use ANS binding as a measure of surface hydrophobicity, but this method is only appropriate in aqueous media. [We attempted to analyse for any changes on protein structure *via* circular dichroism, but this was unsuccessful in our dispersions due to the high absorbance of the oil phase].

It can also be seen in Fig. 4(a) that *G*′′ of the hydrophilic (100% SiOH) silica particles behaves differently compared to the two other particle systems with increasing *γ*. Although all particle types exhibit a plateau in the *G*′′ *versus γ* curve, *G*′′ of the more hydrophobic silica and milled protein particles just decreases after the linear region, whereas the *G*′′ curve of the hydrophilic silica first increases and then decreases at the moduli crossover point. The latter non-linear response has been classified by Hyun *et al.* as weak strain overshoot behaviour.^34^ This is attributed to a temporary structure possessing weak interactions that resist deformation at low *γ* but that are perturbed as *γ* increases. The observed maximum in *G*′′ can be interpreted as an increase in dissipative energy likely due to the partial deformation of aggregated microstructures. As this internal secondary structure breaks, the aggregates of the suspensions align with the flow and the loss modulus decreases. This internal secondary structure can be understood as the reforming of network junctions, facilitated by the highly hydrophilic surface of the silica nanoparticles. Such behaviour has been seen in suspensions of hydrophilic silica in paraffin oil,^23^ group III base oil^19^ and a range of other edible oils.^35^ The non-linear response of the more hydrophobic silica and milled protein particle suspensions suggests that there is less association between aggregates of these particle types and so the network junctions do not significantly reform with respect to structure breakdown during deformation. Consequently, *G*′′ of the latter systems only decreases with *γ* due to a reduction in energy dissipation as the microstructures align with flow. This could be due to less contact points on the particle surface which hinder such interparticle association and structure reformation. Network recovery of hydrophobic silica after application of a large deformation has been shown to be significantly slower than that of hydrophilic silica, owing to their contrasting surface chemistry.^35^

### 3.4 Scaling behaviour of particle gels

In view of the apparent similarities in most of the behaviour of the model silica particles and the protein particles, further analysis of the data was undertaken to see if this could reveal more about the nature of the interactions between the PPI particles in oil, since such a comparison has rarely been made before. The elastic moduli in the LVER of gel networks formed by the three different particle types exhibit a power-law dependence with particle concentration *P* scaling with an exponent *A* (eqn (2)).

2 *G*′ ∝ *P*^*A*^

**Fig. 5:**
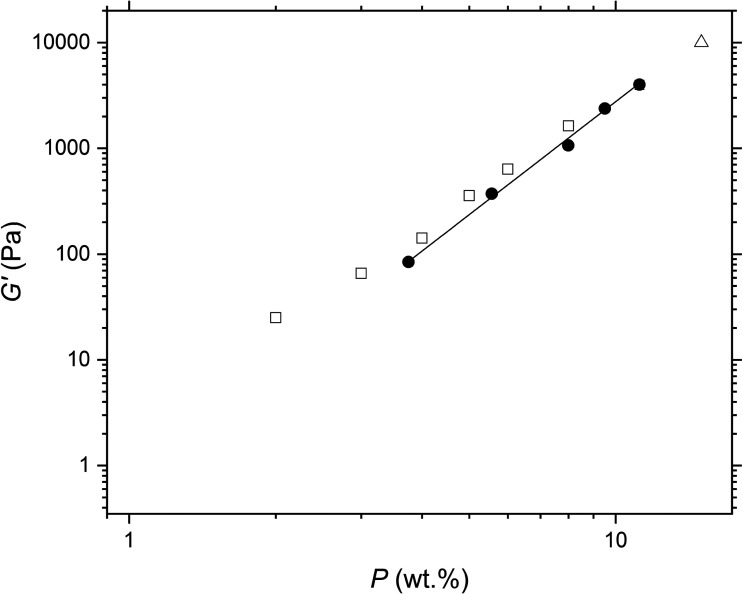
Storage modulus *G*′ as a function of particle concentration *P* for gels of hydrophilic silica in sunflower oil (filled circles). The solid line shows a power law fit to the data with an exponent of 3.55 and an *R*^2^ value of 0.993. For comparison, the *G*′ of hydrophilic silica gels in triglyceride oils taken from the literature are also shown: in olive oil (open triangles) from Patel *et al.*^39^; in sunflower oil (open squares) from Whitby *et al.*^22^

Fig. 5 shows the logarithmic plots of *G*′ *versus P*. This behaviour is typical of colloidal gels in general and has been studied extensively in the literature.^8,28,36–38^ The elastic moduli of the sunflower oil gels structured by the hydrophilic silica had *A* = 3.55, which is consistent with network structures in triglyceride oils shown in other studies.^22,39^ The elasticity of the networks in triglyceride solvents are an order of magnitude lower than those in paraffin oil^23^ and two orders lower than those in mineral oil^20^ at the same particle concentration. For example, at 7 wt%, the elasticity of hydrophilic silica networks in triglyceride, paraffin, and mineral oil was of the order of 10^3^,^22^ 10^4^,^23^ and 10^5^ Pa,^20^ respectively. Whitby *et al.*^22^ suggested that the triglyceride molecules interact with the silica surface *via* H-bonding between the silanol groups and the triglyceride ester carbonyl groups, reducing aggregation and network formation.

**Fig. 6:**
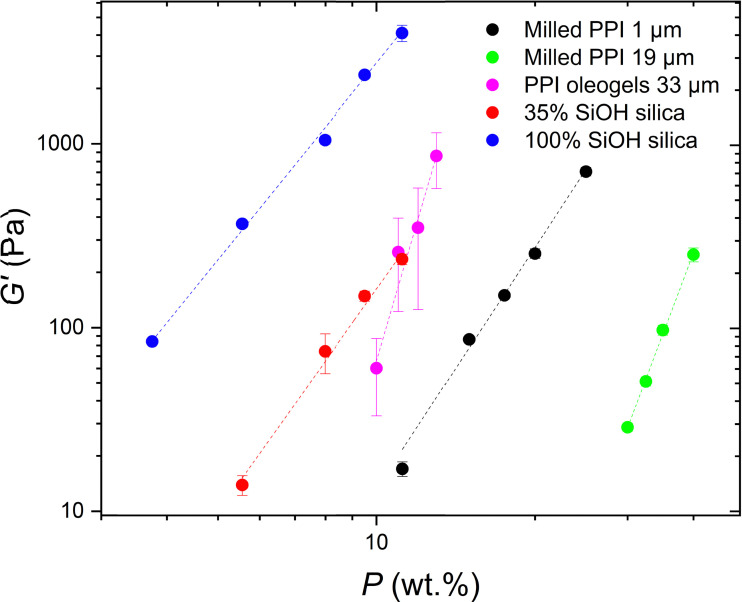
Storage modulus *G*′ as a function of particle concentration *P* of silica and pea protein particle gel networks in sunflower oil: 1 μm μm milled PPI (black); 19 μm milled PPI (green); hydrophobic silica particles with 35% surface silanol coverage (red); hydrophilic silica particles with 100% surface silanol coverage (blue, same data as in Fig. 5); PPI oleogels from Feichtinger *et al.*^28^ (pink). Dashed lines are the fits of the data to the power law model (eqn (2)). Corresponding *R*^2^ values are: black – 0.984, green – 0.996, red – 0.987, blue – 0.993, purple – 0.952. Error bars indicate standard deviation of triplicate measurements.

Again, silica particle gels form networks in sunflower oil at lower concentrations than milled protein particles due to their small particle size. This is clearly illustrated in Fig. 6. Networks of the more hydrophobic silica particles had *A* = 4.03, slightly higher than those of hydrophilic silica. These findings agree with the results of Khan and Zoeller, who reported exponents of approximately *A* = 4 for both hydrophilic and more hydrophobic silica particles in mineral oil.^20^ This value of *A* = 4 is consistent with model predictions of fractal clusters formed by diffusion-limited aggregation,^38^Fig. 6 shows *G*′ *versus* PPI particle concentration for the two PPI particle sizes: PPI with *D*_4,3_ =1 μm (prepared using the smaller scale mill) and PPI with *D*_4,3_ =19 μm (prepared using the larger scale mill – see 2.2.1). The larger PPI particles formed weaker gels than the smaller PPI particles, which again can probably be attributed to the higher specific surface area of the latter. Both protein particle sizes exhibit power-law scaling, but the exponents of the two systems differ from each other and from those of the silica systems. The exponents of the larger and smaller PPI particles are *A* = 7.6 and 4.4, respectively. Thus *A* for the smaller PPI particles is almost the same as with the hydrophobic silica, within experimental error. Possibly this indicates the average ‘surface’ character of the PPI is close to that of the hydrophobic silica.

For comparison, Fig. 6 also shows the scaling behaviour of pea protein particles with an average size of 33 μm reported by Feichtinger *et al.*^28^ In this study, the pea protein particles were prepared by heat treatment in the aqueous phase, causing the protein to unfold and denature, inducing aggregation. The aggregated pea protein particles were then dispersed in the oil phase *via* solvent transfer to create an oleogel system.^28^ In contrast to the ‘native’ protein particles in this work, these oleogel particles exhibit a much higher *A* exponent, forming even stronger gels (higher *G*′) at lower particle concentrations than our milled PPI particles, despite the larger size (quoted as 33 μm) of the particles in the oleogels. Thus, particle size and specific surface area of such protein materials are not the only factors determining the gel strength. Possibly the higher strength of the protein oleogel system is due to more drastic changes in the surface of the protein molecules and their aggregates as a result of their thermal denaturation (in water) and the solvent exchange treatment employed by Feichtinger *et al.*, as opposed to the purely mechanical milling used in this work. The oleogel particles then have more attractive points of contact in the non-aqueous environment, possibly due to a higher degree of surface hydrophilicity. While ball milling can generate high local temperatures^40^ which could potentially cause protein denaturation, we cannot currently confirm whether this occurs in our milled systems. Previous studies have shown that milling PPI in water alters both secondary structure and surface hydrophobicity.^32^ Therefore, it is plausible that milled PPI particles in oil also undergo conformational changes, although it remains uncertain whether these changes result from heat-induced denaturation, mechanical forces during comminution, or a combination of both effects. However, the scaling behaviour in Fig. 6 shows that these two types of PPI particles behave very differently and so it is likely that their internal and surface properties are different. The corresponding exponents for all the systems are given in Table 2.

**Table 2:** Scaling exponent of the power-law dependence of elastic storage moduli *G*′ with particle concentration *P* and fractal dimensions *D*_f_ of non-aqueous gels from silica or pea protein particles. Fractal dimensions were determined using the model of Wu and Morbidelli.^44^ Different superscript letters in the same column indicate a statistically significant difference (*p* < 0.05)

Particle type	*A* (eqn (2))	*B* (eqn (3))	*D*_f_
Milled PPI (*D*_4,3_ = 1 μm)	4.39 ± 0.25^*a*^	−1.69 ± 0.07^*a*^	2.26 ± 0.07^*a*^
Milled PPI (*D*_4,3_ = 19 μm)	7.64 ± 0.22^*b*^	−3.70 ± 0.52^*b*^	2.49 ± 0.07^*b*^
Pea protein oleogels (*D*_4,3_ = 33 μm)^28^	9.8	−2.8	2.71 ± 0.01
Hydrophilic silica (100% SiOH)	3.55 ± 0.11^c^	−0.55 ± 0.05^*a*^	2.33 ± 0.03^*a*^
Hydrophobic silica (35% SiOH)	4.03 ± 0.30^*a*^	−1.55 ± 0.10^*a*^	2.19 ± 0.10^*a*^

**Fig. 7:**
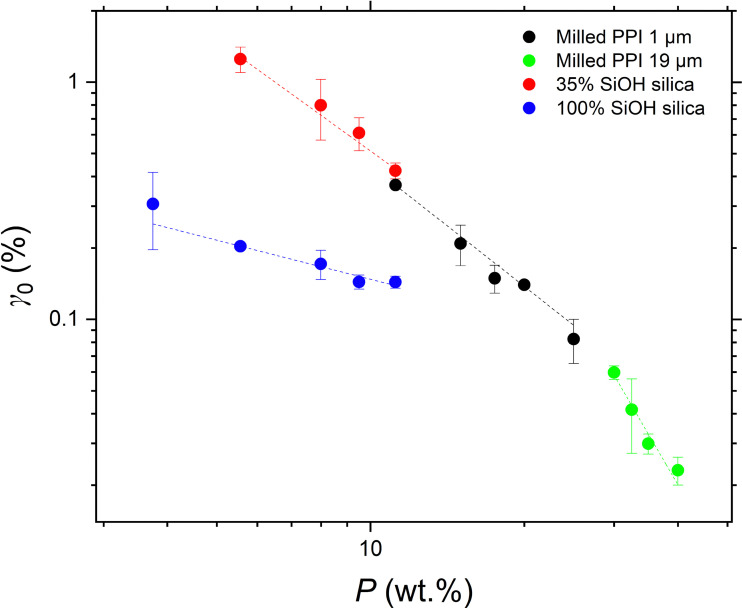
Limit of linearity *γ*_0_ as a function of particle concentration *P* of silica or PPI gel networks in sunflower oil: 1 μm milled PPI (black); 19 μm milled PPI (green); hydrophobic silica particles with 35% surface silanol coverage (red); hydrophilic silica particles with 100% surface silanol coverage (blue). Dashed lines are the fits of the data to the power law model (eqn (3)). Corresponding *R*^2^ values are: black – 0.974, green – 0.963, red – 0.984, blue – 0.973. Error bars indicate standard deviation of triplicate measurements.

Differences in the limits of linearity *γ*_0_ of the particle gel networks were also observed. As was seen with *G*′, *γ*_0_ scales with particle concentration *P* in a power-law relationship with exponent *B* (eqn (3)). *B* was negative for all three particle types – see Fig. 7 and Table 2. This suggests that at higher *P*, the network structures are less effective at dissipating deformation, which could be attributed to the reduced mobility of particles within the denser network. As a result, at higher concentrations, the network yields at lower strains.

3 *γ*_0_ ∝ *P*^*B*^

The PPI network with the smaller particle size had a higher value of *γ*_0_ than that of the larger particle size. The more hydrophobic silica particles formed gels that yield at higher strains than networks of hydrophilic silica at the same particle concentration. This is in contradiction with Yziquel *et al.* who reported that gel networks of hydrophobic silica in paraffin oil were less resistive than hydrophilic silica networks at the same concentration.^23^ However, silica particle network strengths in paraffin oil are reported to be an order of magnitude higher than silica networks in triglyceride oils. Furthermore, the “hydrophobic” silica used by Yziquel has been in fact been found, *via* IR spectroscopy, to have 50% surface silanol groups^20^ – higher than the hydrophobic silica used in this study. The networks formed by the silica particles in both works may therefore differ in density and microstructure, giving rise to different absolute yielding strains. Despite this, the trends remain consistent, with a larger, more negative exponent between particle concentration and yielding strain observed for silica particles of higher hydrophobicity.

In principle, scaling theories can be used to relate the macroscopic rheological properties of colloidal gels to microscopic structural parameters. In these theories the gel network is usually considered as particle flocs possessing fractal structure, *i.e.*, the structure remains invariant, or ‘self-similar’ across different length scales.^41^ These models have been applied extensively to silica particle gels in the literature.^23,42,43^ Although a variety of models have been reported, the approach is consistent: indirect calculation of the fractal dimension (*D*_f_) from the relationship between rheological measurements, such as *G*′ or *γ*_0_, with particle concentration.^42,44,45^ Since the milled protein particles are formed of tightly bound protein aggregates and are not strictly monodisperse (see Fig. 2), networks of these particles may not display self-similarity on a sufficiently large length scale to be considered fractal. These theories can, however, be applied to indicate differences in the microstructure of networks of the aggregates themselves.^28^

To characterize the network structure of the silica and protein particles in oil the model of Wu and Morbidelli^44^ has been applied (see results in Table 2). Wu and Morbidelli derived the two exponents *A* and *B* as:4
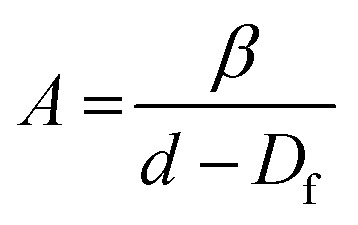
5
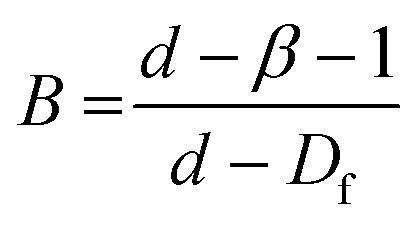
where *d* is the Euclidean dimension of the system and *β* is a constant which accounts for the elastic contributions of the intrafloc and backbone of the network. Eqn (4) and (5) can be combined to eliminate *β*, which leads to an expression of *D*_f_ as a function of both rheological scaling exponents:6
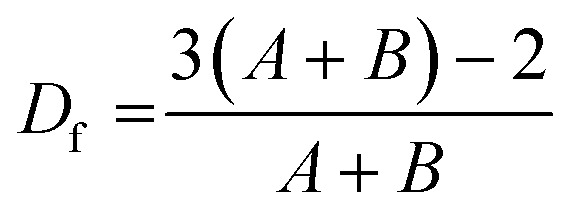


As usual, a fractal dimension closer to 3 indicates a denser structure, a value closer to 2 suggests a floc structure that is more diffuse. Using this model, it was found that the data for the 1 μm PPI particles gave a lower fractal dimension than the 19 μm particles, *i.e.*, the PPI particle flocs are more open and diffuse for the smaller particles. The more dense structure of the larger particles gives rise to the lower observed limit of linearity, since as the network gets denser it becomes less mobile, leading to more localized failure rather than distribution of the deformation throughout the network.

In a similar fashion, when the Wu and Morbidelli model^44^ was applied to the silica data, a slightly higher fractal dimension was obtained for the more highly aggregating hydrophilic silica than the more hydrophobic silica, although this was within experimental error. Whitby *et al.*^22^ studied silica systems in olive oil *via* confocal microscopy and found that reducing the silanol content of silica particles by 50% caused the pore size to double. This was attributed to weaker interactions between the more hydrophobic particles, giving rise to a more open, branched network structure.

### 3.5 Yield stress analysis

**Fig. 8:**
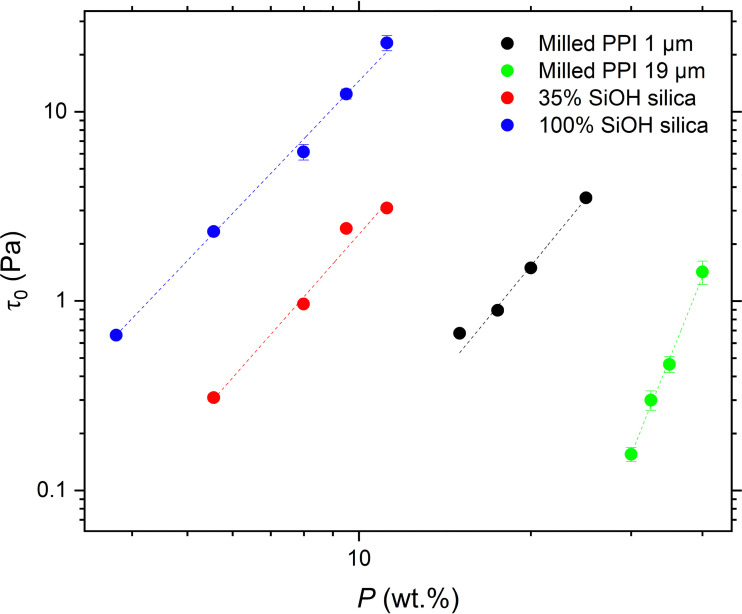
Yield stress (*τ*_0_) as a function of particle concentration (*P*) of silica or PPI gel networks in sunflower oil: 1 μm milled PPI (black); 19 μm milled PPI (green); hydrophobic silica particles with 35% surface silanol coverage (red); hydrophilic silica particles with 100% surface silanol coverage (blue). Error bars represent the standard error in the shear stress values derived from the Herschel–Bulkley model (eqn (7)). Dashed lines are the fit of the data to a power law model (eqn (8)) with the corresponding exponents detailed in Table 3. Corresponding *R*^2^ values are: black – 0.991, green – 0.991, red – 0.952, blue – 0.993.

An alternative way of analysing the rheological data is to consider the systems as viscoelastic gels with specific yield stress values. The yield stress values calculated from the flow curves of the particle systems are plotted as a function of particle concentration *P* in Fig. 8. The yield stress values (*τ*_0_) were obtained by application of the Herschel–Bulkley model (eqn (7)) to the stresses (*τ*) measured with increasing *γ̇*:

7 *τ* = *τ*_0_ + *kγ̇*^*n*^

where *k* is the consistency index and *n* is the flow index.

All particle types exhibit a power-law scaling and Piau *et al.*^42^ proposed a model relating the yielding behaviour of particle gels to the fractal dimension of their network structure. The model defines this relationship as:8
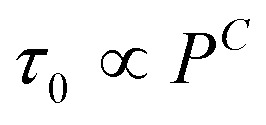
where the exponent *C* is defined as:9
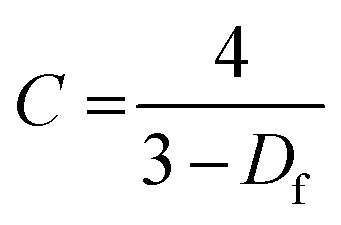


This model is based on the relationship between the correlation length (*ξ*) and the energy stored in the polymer network, which equals the applied stress energy at the yield point. As the yielding of the network is related to the rupturing of the backbone, the dimensionality predicted by the Piau model is likely to be representative of the structure of this backbone rather than the aggregates within the flocs. The fractal dimensions predicted by the Piau model of the 1 μm PPI particles and both silica particle systems, shown in Table 3, are lower than those predicted by the model of Wu and Morbidelli (Table 2). This suggests that the network backbones of the protein and silica systems are more open compared to the particle flocs overall. Similar behaviour has been observed for capillary suspensions of milled aluminium oxide particles in paraffin oil,^38^ and zein oleogel particles in soybean oil.^46^ Bossler *et al.* reported that as the particle size of the aluminium oxide was reduced, the fractal dimension of the network decreased − these authors also found that the Piau model predicted lower fractal dimensions than that of Wu and Morbidelli. This was attributed to an inhomogeneous microstructure of the capillary suspensions with a higher local solid volume fraction inside of the flocs compared to the backbone.^38^ The backbone fractal dimension of the 1 μm PPI particles is comparable to that of the hydrophobic silica within experimental error, further indicating that the two systems exhibit similar interactions.

**Table 3:** Scaling exponents (*C*) of the power-law dependence of yield stress (*τ*_0_) with particle concentration (*P*) and fractal dimensions (*D*_f_) of non-aqueous gels from silica or pea protein particles as determined *via* the model of Piau *et al.*^42^ Different superscript letters in the same column indicate a statistically significant difference (*p* < 0.05)

Particle type	*C* (eqn (3))	*D*_f_
Milled pea protein (*D*_4,3_ = 1 μm)	3.67 ± 0.23^*a*^	1.91 ± 0.07^*a*^
Milled pea protein (*D*_4,3_ = 19 μm)	7.51 ± 0.36^*b*^	2.47 ± 0.03^*b*^
Hydrophilic silica (100% SiOH)	3.15 ± 0.08^*c*^	1.73 ± 0.03^*c*^
Hydrophobic silica (35% SiOH)	3.41 ± 0.35^*a*,*c*^	1.83 ± 0.12^*a*^

In contrast, for the 19 μm PPI particles the same fractal dimension was predicted by both models, suggesting both a very dense network backbone and particle flocs, in other words, a more homogenous network with an indistinguishable backbone and flocs.

### 3.6 Confocal laser scanning microscopy

**Fig. 9:**
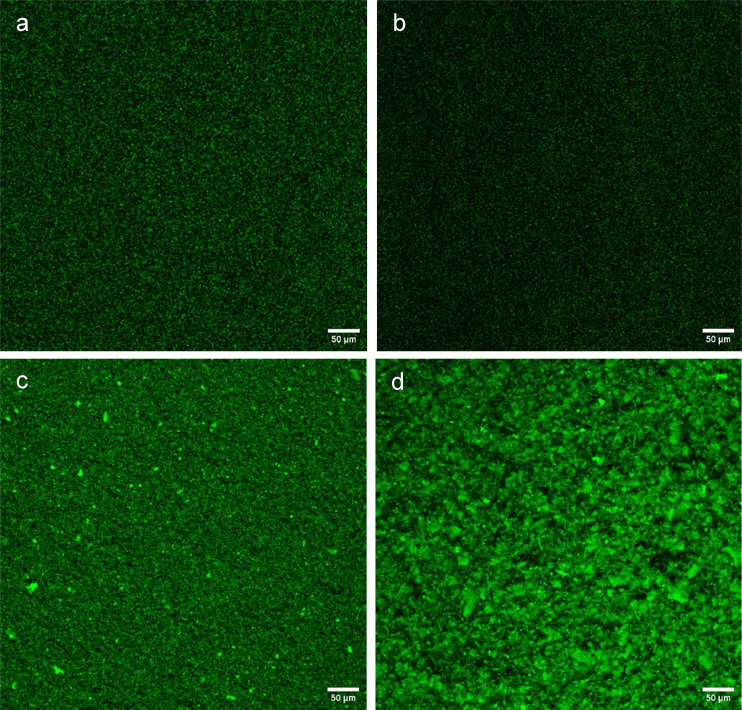
Confocal microscopy images of sunflower oil dispersions of (a) hydrophobic and (b) hydrophilic silica at 3.75 wt% and milled pea protein particles with a size of (c) 1 μm and (d) 19 μm at particle concentrations of 11.2 and 35 wt% respectively. Images had non-linear and brightness adjustments to ensure both small and large protein aggregates were visible. A soften filter was also applied *via* ImageJ. This correction method was consistent across all images.

The way to try and confirm directly the structure, fractal dimensions, *etc.*, of particle gels is *via* analysis of microscopic images of the networks. However, this is not simple, requiring high quality images of gels not distorted by preparation of the samples for the microscopy. Confocal laser scanning microscopy (CLSM) simply requires adequate labelling of the relevant components either before or after gel formation and was employed to try and confirm the structural features predicted by the above rheological analysis. Firstly, the resulting CLSM images (Fig. 9) clearly confirmed the size differences amongst the various particle types. The smaller PPI particle system shows the presence of a few larger particles or agglomerates within the network (Fig. 9(c)), reflecting the bimodality of the PSD noted earlier (Fig. 2). The larger PPI particles showed a unimodal size distribution and the particles in the CLSM images are correspondingly seen to be more homogenous in size (Fig. 9(d)). The individual silica nanoparticles are too small to be distinguished by CLSM but clearly they form much smaller aggregate structures than the protein (Fig. 9(a) and (b)). Insights into the direct fractal dimension of the particle networks were not possible with the CLSM images shown.

### 3.7 Pre-wetted particles

**Fig. 10:**
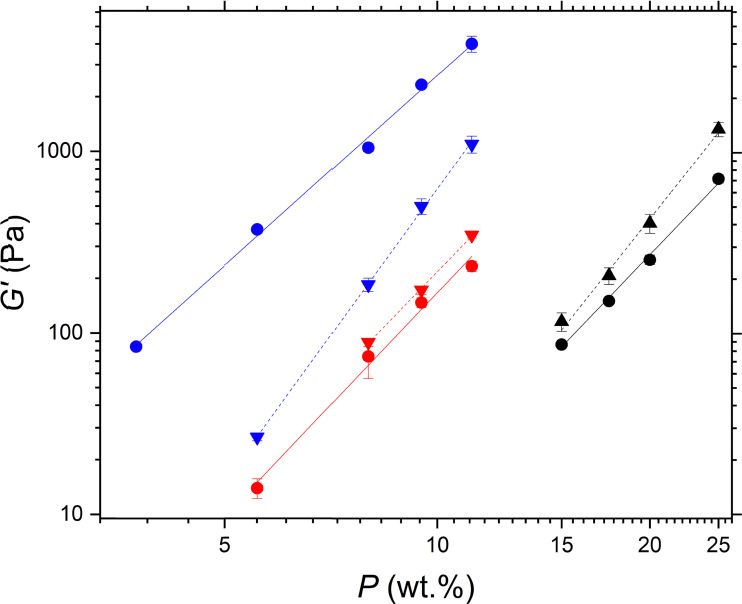
Storage modulus (*G*′) as a function of particle concentration (*P*) of non-aqueous gels from silica and PPI with and without pre-wetting the particle surface prior to dispersion. Blue symbols = 100% SiOH silica, red symbols = 35% SiOH silica and black symbols = milled PPI 1 μm. Solid lines indicate the power law model fits (eqn (2)) for non-wetted particles, while dashed lines represent the fits for the wetted particles. Corresponding *R*^2^ values for pre-wetted samples are: black – 0.987, red – 0.999, blue – 0.998.

The effect of pre-wetting the colloidal particles with a solvent of a higher polarity prior to dispersion in the oil phase was explored. This technique has been shown to alter the surface properties of such particles through molecular adsorption,^47–49^ with the potential to act as a surfactant.^50^ Ethanol was selected on account of its polarity, hydrogen-bonding ability, volatility, and food-grade classification.^51^ Pre-wetting the hydrophilic silica particle surface with ethanol reduced the elastic moduli at a given particle concentration, *i.e.*, gave weaker gels (Fig. 10). This is presumably due to the solvent hindering network formation *via* the hydroxyl group of ethanol H-bonding to the surface Si–OH, with the ethyl chain pointing outwards, thus imparting greater surface hydrophobicity and compatibility with the oil. At the same time, H-bonding between Si–OH groups on different particles is disrupted. The scaling exponents (see Table 4) increased from 3.55 to 5.38, suggesting a marked change in the particle–particle interactions and structure of the network. In contrast, but as expected, pre-wetting the already more hydrophobic silica particles had minimal effect on the gel network strength and scaling exponent. This is corroborated by literature results which show that ethanol has the propensity to hydrogen bond with surface silanol groups to form clusters, whereas such clusters are not formed on a hydrophobic silica surface.^49^ Although ethanol is the most frequently used solvent to aid dispersion of such particles in oil, we also performed a few preliminary tests with acetone and butanol. Butanol gave similar results to ethanol, but acetone had less effect, probably because it is a hydrogen bond acceptor only.

**Table 4:** Scaling exponents of the power-law dependence of elastic storage moduli and the limit of linearity with particle concentration of non-aqueous gels from silica or pea protein particles pre-wetted with ethanol prior to dispersion. Fractal dimensions were determined using the model of Wu and Morbidelli.^44^ Different superscript letters in the same column indicate a statistically significant difference (*p* < 0.05)

Particle type	*A* (eqn (2))	*B* (eqn (3))	*D*_f_
Milled PPI (*D*_4,3_ = 1 μm)	4.08 ± 0.17^*a*^	−1.49 ± 0.44^*a*^	2.23 ± 0.14^*a*^
Milled PPI pre-wet ethanol (*D*_4,3_ = 1 μm)	4.93 ± 0.26^*b*^	−1.79 ± 0.01^*a*^	2.36 ± 0.05^*a*^
Hydrophilic silica (100% SiOH)	3.55 ± 0.11^*c*^	−0.54 ± 0.06^*a*^	2.34 ± 0.03^*a*^
Hydrophilic silica (100% SiOH) pre-wet ethanol	5.38 ± 0.06^*d*^	−2.24 ± 0.43^*a*^	2.36 ± 0.09^*a*^

PPI particles pre-wetted with ethanol formed stronger networks than those without pre-wetting, despite having comparable particle sizes to unwetted PPI particles, suggesting that surface-bound ethanol increased particle–particle interactions. As already mentioned, the protein particle ‘surface’ is far more complex and indistinct than that of the silica and there are many protein functional groups that could interact and bind to ethanol molecules, altering the overall surface properties of the particles. When the Wu and Morbidelli model was applied as described above, the results suggested that the pre-wetting had minimal effect on the density of the particle networks – see Table 4. This highlights the potential of pre-wetting as technique to modify interparticle behaviour of both simple and more complex particles.

## 4. Conclusions

In this paper, the rheological properties of pea protein isolate (PPI) particles dispersed directly in oil have been reported. The PPI particles consist of large, tightly bound aggregates which require strong mechanical treatment by milling to break them down to micron and sub-micron particle sizes in oil. PPI particle behaviour was compared in detail with two types of model silica particles with known surface chemistry: hydrophilic and hydrophobic silica. All three particle types form structured aggregates in sunflower oil which form a space-spanning network at sufficiently high particle concentrations. As the size of the protein particles decreased, the degree of aggregation was greater, shown by higher *η* and gel network strength (*G*′). Despite the amphiphilic nature of PPI, milled PPI particles form weaker networks than both types of silica particles, likely due to their larger particle size and therefore lower specific surface area and larger interparticle distances. Surface complexity of the protein may also play a role, leading to less contact points between neighbouring protein particles.

Scaling theories were applied to both the elastic modulus and yield stress data of the different particle systems. The fractal dimensions estimated from the elastic moduli, using the model of Wu and Morbidelli,^44^ were higher than those obtained from the yield stresses using the Piau model.^42^ The former is a model of the structure within the particle flocs, whereas the latter is a model describing the dimensionality of the network backbone. The differences in fractal dimensions between the two models were attributed to a network structure with a higher local solid volume inside the flocs compared to a less dense backbone, similar to capillary particle suspensions in oil.^38,46^ For the network of larger PPI particles, the two fractal dimensionalities were indistinguishable, suggesting a uniform, high solid volume throughout. Although the two models predicted different fractal dimensions the trends were consistent: as particle size decreased the fractal dimension decreased, indicating a more open network structure. Networks of the smaller milled PPI particles (1 μm) had almost the same inter-floc and intra-floc fractal dimensions as the hydrophobic silica, suggesting that the average ‘surface’ character of the PPI may be close to that of the silica. The particle–particle interactions of the milled protein and hydrophilic silica could be altered by pre-treating the particle surface with a high polarity solvent, leading to changes in network elasticities. Further microscopy work would help to confirm these conclusions, though this is difficult without introducing artefacts. Overall, the similarities in behaviour of the very different types of particles when dispersed in oil is quite remarkable, so that a greater understanding and control of the rheology of the protein-based systems seems possible, which could have many applications in various consumer, pharmaceutical and agrochemical products, for example.

## Conflicts of interest

There are no conflicts to declare.

## Supplementary Material

SM-021-D5SM00596E-s001

## Data Availability

Data will be made available on request. Supplementary information is available. See DOI: https://doi.org/10.1039/d5sm00596e.
