# Supplementary material for: Dispersion behaviour of insoluble particles with different surface properties in non-aqueous media – biopolymer based oleogels
Source: Soft Matter. 2025 Aug 29;21(38):7460–75. doi: 10.1039/d5sm00596e (PMC12415833; doi:10.1039/d5sm00596e)
Supplement: SM-021-D5SM00596E-s001 [file SM-021-D5SM00596E-s001.pdf]

Supplementary Information (SI) for Soft Matter.
This journal is © The Royal Society of Chemistry 2025

Dispersion behaviour of insoluble particles with different surface properties in non-aqueous media – biopolymer based oleogels

Supplementary information

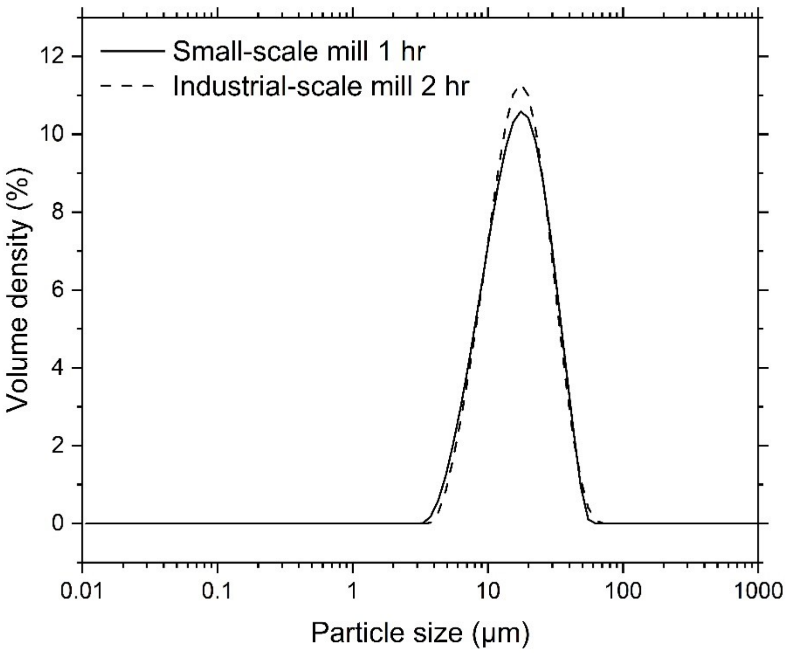

Figure S1. Particle size distribution for milled pea protein isolate particles in sunflower oil, prepared using small-scale and industrial milling apparatus. Samples processed with the small-scale mill were milled for 1 hour (solid lines), while those prepared using the industrial-scale mill were milled for 2 hours (dashed lines).
